# Pathobiologic Markers of the Ewing Sarcoma Family of Tumors: State of the Art and Prediction of Behaviour

**DOI:** 10.1155/2011/856190

**Published:** 2010-10-14

**Authors:** Alfredo Pinto, Paul Dickman, David Parham

**Affiliations:** ^1^Calgary Laboratory Services, University of Calgary, Alberta Children's Hospital, 2888 Shaganappi Trail NW, Calgary, AB, Canada T3B 6A8; ^2^Department of Pathology, Phoenix Children's Hospital, 1919 E. Thomas Road, Phoenix, AZ 85016, USA; ^3^Departments of Pathology and Pediatrics, University of Arizona, College of Medicine, Phoenix, AZ 85016, USA; ^4^Health Sciences Center, University of Oklahoma, Oklahoma City, OK 73104, USA

## Abstract

Over the past three decades, the outcome of Ewing sarcoma family tumor (ESFT) patients who are nonmetastatic at presentation has improved considerably. The prognosis of patients with metastatic disease at the time of diagnosis and recurrence after therapy remains dismal. Drug-resistant disease at diagnosis or at relapse remains a major cause of mortality among patients diagnosed with ESFT. In order to improve the outcome for patients with potential relapse, there is an urgent need to find reliable markers that either predict tumor behaviour at diagnosis or identify therapeutic molecular targets at the time of recurrence. An improved understanding of the cell of origin and the molecular pathways that regulate tumorigenicity in ESFT should aid us in the search for novel therapies for ESFT. The purpose of this paper is thus to outline current concepts of sarcomagenesis in ESFT and to discuss ESFT patterns of differentiation and molecular markers that might affect prognosis or direct future therapeutic development.

## 1. Introduction

Since James Ewing in 1921 first described a diffuse hemangioendothelioma of bone [1], several studies have attempted to elucidate the histogenesis of Ewing's sarcoma (ES) [2, 3]. Based on histology, electron microscopy, and immunostains, it was postulated that ES represents a primitive mesenchymal neoplasm with the potential for multidirectional differentiation [2]. Neural and neuroectodermal differentiation was demonstrated in cell lines [4, 5] and tissue sections [6, 7], and tumors with the histology of Ewing sarcomas were described in soft tissue [8–10]. Lesions with atypical features also appeared [11]. Askin et al. [12] described a distinctive neoplasm in the chest wall that we now recognize as belonging to this group of cancers. Originally, neuroectodermal tumors of bone [13] and soft tissue [8] were described as a separate entities, but some overlap became apparent [14]. The term “primitive neuroectodermal tumor” (PNET) was given to these latter lesions, and the sobriquet “peripheral PNET” (pPNET) was later applied to separate them from unrelated lesions of the central nervous system [15]. With the finding of specific translocations t(11;22) and t(21;22) and accompanying genetic fusions, it became generally accepted that ES of bone and soft tissue, pPNET of bone and soft tissue, Askin's tumor, and ES with neural differentiation represent a single tumor entity with common antigenic profiles, cytogenetic aberrations, and protooncogene expression. This common entity, which has been designated the Ewing sarcoma family of tumors (ESFT), possesses a limited capacity for several different degrees of differentiation [16–18]. 

The cell of origin for ESFTs has been hotly debated ever since their discovery [16, 19, 20]. Candidates have included neuroectodermal and mesenchymal cells of hematopoietic and nonhematopoietic origin. Human marrow mesenchymal stem cells (MSCs) currently appear to be a strong candidate as the cell of origin of ESFT. MSCs are permissive for the characteristic *EWS-FLI1* fusion and can initiate reprogramming toward ES cancer stem cells [20, 21]. 

The overall prognosis of ESFT has improved with the improved use of chemotherapy for localized tumors. However, metastatic and recurrent disease still has a dismal prognosis. A number of pathological and molecular characteristics have been linked to the clinical outcome of ESFT. In this paper, we will outline the pathological features of ESFT, review the current concepts about their sarcomagenesis, discuss their capacity for multipotential differentiation, and focus on the controversies and impact of neural differentiation and other pathobiological parameters in patient outcome.

## 2. Morphology

Before cytogenetics established that ES and pPNET share the balanced translocation t(11;22) (q24;q12) in over 90% of cases [22], ES and pPNET were regarded as totally separate entities distinguished by their morphologic and ultrastructural characteristics. ES occurred primarily in bone and pPNET in soft tissue. However, these boundaries crumbled with reports of discrepant features, such as an extraskeletal neoplasm resembling ES [9], a neuroectodermal tumor of bone [13], and ES with neural features [14]. Thanks to cytogenetic and molecular studies, it is now accepted that ES and pPNET comprise opposite poles of a histological continuum of a single group of neoplasms. In between lie lesions with varying degrees of atypical features (atypical ES) and differentiation. With the use of genetic tools, evidence emerged that morphological and immunohistochemical spectrum of ESFT may be broader than has traditionally been accepted [23], and additional histological patterns of ESFT have now been described [23, 24]. The histology of ESFT can be described as “classical”, “atypical”, or “variant”. The following section will detail these histological features.

### 2.1. Classical Ewing Sarcoma Histology

**Figure 1:**
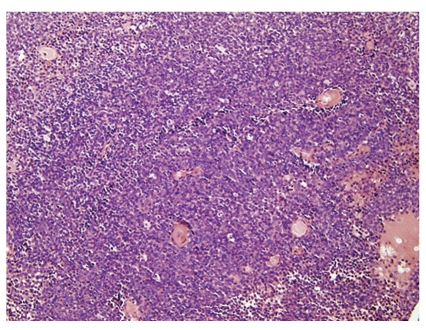
Classical Ewing's sarcoma, microscopic image of typical histomorphology. The lesion comprises patternless sheets of small blue cells with round, regular nuclei, even nuclear margins, minimal cytoplasm, and indistinct boundaries.

Microscopically, classical (typical) ES is the prototypic undifferentiated small round cell tumor, composed of sheets of monotonous round or oval cells with primitive-appearing nuclei and moderate amount of clear to amphophilic cytoplasm (Figure 1). The nuclear characteristics of the prototypic ES are those of a primitive uncommitted cell, that is an oval configuration with smooth contours, single inconspicuous nucleoli, and finely dispersed chromatin. The nuclei are generally centrally located and surrounded by a thin rim of clear to vacuolated cytoplasm. The lesions are highly cellular with little intervening intercellular space. Paradoxically, typical cases contain few mitoses. Cytologically, these tumors comprise a mixture of lightly staining “primary” cells and darkly staining “secondary” cells. Some feel that the “secondary” cells are effete versions of the “primary cells” that result from degenerative changes that lead to nuclear hyperchromasia or basophilia, but some ultrastructural studies show no signs of degenerative changes [25]. Sixty to seventy-five per cent of ESFTs [23, 24] are reported to have this classical histology (Table 1).

### 2.2. Variant Histologies

#### 2.2.1. Atypical ES (or Large Cell ES)

The cells of atypical ES are larger and more pleomorphic. They contain pleomorphic nuclei with indented, irregular nuclear membranes, conspicuous nucleoli, and increased amounts of cytoplasm that is often more eosinophilic than clear. The cells may contain abundant intracytoplasmic glycogen that gives them a clear appearance. A spindle-like configuration with fusiform nuclei can affect more than 50% of the tumor field. Atypical variants with epithelioid or clear cells [24] and a synovial-like pattern have been described [23]. Coincident with the appearance of atypical features, increased mitotic activity appears. Although frank neural differentiation by definition should be lacking, subtle signs may appear by electron microscopy or single immunostains.

#### 2.2.2. pPNET

**Figure 2:**
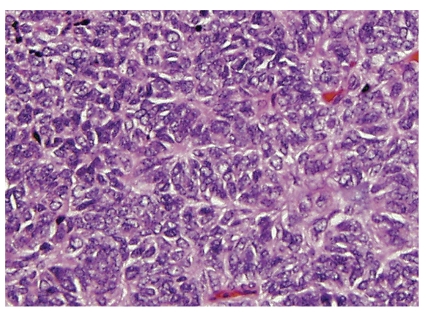
Peripheral primitive neuroectodermal tumor with Homer Wright rosettes. The rosettes contain a circular wreath of oval nuclei that surround a pale eosinophilic, fibrillary core.

One of the best descriptions of pPNET was written by Jaffe et al. [13], who described neuroectodermal tumor of bone with prominent Homer Wright rosettes (previously called “malignant neuroepithelioma” or “peripheral neuroepithelioma”). They proposed this entity to be a distinct neoplasm, although the Intergroup Ewing Sarcoma Study had included rosette-forming lesions as a form of ES [26]. Jaffe et al. noted that no attempt had been made in prior publications to distinguish Homer Wright rosettes from perivascular or apoptotic pseudorosettes, and they postulated that Homer Wright rosettes are a marker for pPNET. This observation was later confirmed by Llombart-Bosch and Schmidt [7, 27]. As per the original description of neuroblastoma by James Homer Wright, these rosettes should consist of a closely apposed circle of cells with a peripheral wreath of nuclei and a central core of neuropil (Figure 2). The number of rosettes found in pPNETs varies from isolated to numerous. If isolated, they generally contrast with the background of monotonous fields of small round blue cells, but at times the histology can be subtle. Lesions are termed pPNET independent of the degree of differentiation and the number of rosettes. Jaffe presciently proposed that ES may be the most undifferentiated form of pPNET. Subsequently, several retrospective studies indicated that by immunohistochemistry features of neural differentiation can also be revealed in ES, using antibodies against proteins such as neuron-specific enolase, Leu 7 (later termed CD57), and S100 [7, 28, 29]. In 1991 Schmidt et al. [27] proposed that pPNET could be defined as tumors having Homer-Wright rosettes or expressing two different neural immunomarkers. Their retrospective study, which covered several decades of treatment, indicated that pPNETs had a worse clinical outcome than other forms of ESFT. Using Schmidt's criteria, the reported incidence of pPNET varies from 12% to 23%, compared to other ESFTs (Table 2).

#### 2.2.3. Adamantinoma-Like Pattern

This is a recently described variant of ESFT described by Bridge et al. as an example of “phenotypic drift” [30]. This pattern comprises well-formed nests of moderately hyperchromatic cells with striking peripheral palisading and a prominent host desmoplastic response. Adamantinoma-like ESFTs are positive for CD99, FLI1, pan-cytokeratin, and in distinction from other forms of ESFT, high molecular weight cytokeratin [23]. Note that cytogenetic features characteristic of ESFT distinguish these lesions from true adamantinoma of bone [30, 31]. 

#### 2.2.4. Spindle Cell Sarcoma-Like Pattern

This is another recently described pattern of ESFT, composed of intersecting fascicles of spindle cells. It should however be noted that brief mention of a similar lesion appeared in 1983 in the description of “peripheral neuroepithelioma” in the first edition of Enzinger and Weiss's classic textbook [32]. Rare cases were also described in 1992 by Cavazzana et al. [33], although genetic confirmation was lacking at that time. There are well-developed, branching intratumoral blood vessels, reminiscent of hemangiopericytoma-like spindle cell sarcomas such as synovial sarcoma. CD99 and FLI1 are positive in all cases, and pan-cytokeratin is positive in two-thirds. Confirmatory genetic testing is required for this diagnosis (see below).

#### 2.2.5. Sclerosing

This new pattern is described as composed of abundant hyalinised matrix. Two cases described by [23] were felt to be reminiscent of sclerosing epithelioid fibrosarcoma or sclerosing rhabdomyosarcoma. The neoplastic cells generally resemble the cells of typical ESFT. CD99 and FLI1 were positive in all cases, pan-cytokeratin was positive in 2 cases, and all were negative for high molecular weight cytokeratin. This lesion must be distinguished from desmoplastic small round cell tumor (DSRCT), which has a different cytogenetic aberration. However, rare tumors may resemble DSRCT histologically and immunophenotypically but contain cytogenetic features typical of ESFT [34].

#### 2.2.6. Vascular-Like

Similar to Ewing's original report of “hemangioendothelioma of bone”, the observation that some ESFTs resemble vascular lesions persists. In Llombart-Bosch's series [24], there were 9 such cases (2%). ESFTs with this pattern contain pseudoendothelial elements that produce angiomatoid spaces lined by elongate tumor cells surrounding lacunae filled with erythrocytes and plasma. Unlike true vessels, the cells lining the surface of the spaces lack basal lamina and are in continuity with the surrounding typical ES. Of note, structures resembling Weibel-Palade bodies have been described in some ultrastructural descriptions [35], although genetic confirmation was lacking in these early reports.

### 2.3. Current Morphological Controversies

**Figure 3:**
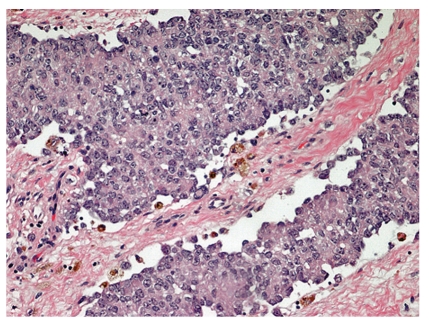
Atypical Ewing sarcoma, postchemotherapy and recurrence. This recurrent ESFT displays epithelioid features, such as increased eosinophilic cytoplasm and cohesive nests.

Two large recent retrospective series of genetically confirmed ESFT cases [23, 24] outline the heterogeneity of histological patterns in these lesions (Table 1). It is unclear in these reports whether the patterns were homogeneous or mixed in individual tumors, and current limited pretreatment biopsies preclude extensive histological assessment. In one of the series [24], all the unusual histological subtypes were grouped under the rubric of “atypical variant”. Patterns described as synovial-sarcoma-like, sclerosing, and adamantinomatous were not considered independent entities. Noteworthy is that unlike Llombart-Bosch et al., the Folpe series did not report any hemangioendotheliomatous cases, further emphasizing their rarity as a subtype. Questions remain as to whether or not unusual patterns may be seen in combination with the classical pattern and whether the morphological phenotype of recurrent tumors can be modified by chemotherapy. In our experience, we have seen one case of classical ESFT histology that recurred with an atypical epithelioid histological pattern (Figure 3). Others have described lesions with ganglioneuroma-like features, both before [36] and after [37] chemotherapy.

## 3. Immunohistochemistry

Traditionally, the diagnosis of ESFT has been made by exclusion, but this situation considerably improved over the last 20 years with the introduction of new immunohistochemical markers. These markers include CD99 [38], FLI1 [39], and caveolin1 (CAV1) [24], which are commonly expressed in ESFTs and differentiate them from other small round cell tumors (Table 2). In addition, markers of neural, epithelial, and mesenchymal differentiation have been described in various subsets of ESFT. In the following section, we will briefly describe the immunohistochemical markers associated with ESFT.

### 3.1. CD99

CD99 is a membrane associated protein that is closely related to the murine THY-1 antigens, major cell surface glycoproteins of murine brain, and thymus cells [40]. It is a 32 KDa membrane glycoprotein that is highly expressed in most cases of ESFT [41]. This protein is encoded by a pseudoautosomal gene found in both X and Y chromosomes. CD99 is also known as MIC2 and is recognized by monoclonal antibodies 12E7, HBA71, O13, and HO36-1.1. It has a key role in several biological processes, including: cell adhesion, migration, and apoptosis; differentiation of T thymocytes; diapedesis of lymphocytes to inflamed vascular endothelium; maintenance of cellular morphology; and regulation of intracellular membrane protein trafficking [42, 43]. Modulation of CD99 expression significantly modifies cell growth in anchorage-independent conditions, and it affects cell migration, tumorigenesis, and metastatic activity in other models.

In ESFT, CD99 appears to prevent terminal neural differentiation [44]. This process is dependent on changes in mitogen-activated protein kinase pathway signalling. [44]. CD99 contributes to cell proliferation, migration, and metastasis of ESFT cells [44]. Questions remain as to whether CD99 is a downstream target of EWS/FLI1.

### 3.2. FLI1

As will be described below in more detail, the reciprocal translocation t(11;22) results in juxtaposition of the amino terminal domain of *EWS* to the carboxyl terminus of *FLI1* [45]. *FLI1* is normally expressed in endothelial cells and hematopoietic cells, including T lymphocytes. It is a member of the ETS (erythroblastosis virus-associated transforming sequences) family of DNA-binding transcription factors, and it is involved in cellular proliferation and tumorigenesis [46]. Folpe et al. [39] showed that a polyclonal antibody to the carboxyl terminus of FLI1 protein was a relatively sensitive (71%) and highly specific (92%) marker of ESFT. 

These results and those of Nilsson et al. [47] indicate that FLI1 antibodies may play a valuable role in the immunohistochemical diagnosis of small blue round cell tumors. The combination of CD99 and FLI1 immunostaining appears to improve the specificity of these markers for diagnosis of *EWS/FLI1* fusion-positive ESFT [48].

### 3.3. Caveolin

Caveolae are plasma membrane invaginations that regulate several intracellular signaling pathways. The defining components of caveolae are from 21 to 24 kDa molecules termed caveolins (CAV). There are several types of caveolins. CAV-1 and CAV-2 are ubiquitously expressed, whereas CAV-3 is only expressed in muscle tissue [49]. CAV-1 is the only member of the family required for caveolar formation. CAV-1 acts as a tumor suppressor in breast cancer and other tumors [50], and it is a direct *EWS-FLI1* transcriptional target [51]. In ESFT, CAV-1 is a key determinant of tumorigenicity, which implies that it may be a molecular target for new therapeutic strategies [51]. It has also been proposed as a potential diagnostic marker for ESFT [24], but this observation needs additional study for confirmation.

### 3.4. Markers of Neural Differentiation

As noted above, a neural phenotype has been detected in many ESFT by immunohistochemistry and ultrastructure. In 1982, Schmidt et al. [14] noted that neural features such as processes, dense core granules, and microtubules could be noted in cases diagnosed as Ewing sarcoma. In 1986, Dickman and Triche [52] established diagnostic criteria for differentiation of extraosseous Ewing sarcoma (EOE) versus primitive rhabdomyosarcoma by electron microscopy. In a study of EOE treated with Intergroup Rhabdomyosarcoma Study protocols, Shimada et al. [53] identified 14 cases as pPNET based on neural and schwannian differentiation detected by immunohistochemistry and ultrastructure. 

Neural differentiation can be demonstrated in ESFT by antibodies against neuron-specific enolase (NSE), CD57 (Leu-7, HNK1), S-100 [28, 29], neurofilaments (NF), and synaptophysin [54]. Schmidt et al. [27] proposed that pPNET diagnosis could be based on two different neural markers; before their study, a diagnosis of pPNET was made on only one neural marker (NSE, Leu-7, S-100, NF). Partial neural differentiation appears to be a frequent event in ESFT, judging from immunohistochemical and electron microscopic studies [54]. 

Both ES and pPNET are forms of ESFT, differing only in extent of neuroectodermal phenotype and morphological differentiation [18]. Literature review of older publications indicated an overall survival of only 30% for PNET and 65%–70% for EOE [16].

### 3.5. Miscellaneous Markers of Differentiation in ESFT

Positivity for various other phenotypic markers has been reported in ESFT. Markers such as desmin [55] and cytokeratins [23, 24] have been described. CD99-negative small round cell tumors with polyphenotypic expression have been reported [56]. Katz et al. [34] reported a polyphenotypic intraabdominal neoplasm with features of both ESFT and DSRCT and *EWS/FLI1* transcript. Thorner et al. [56] described a DSRCT-like polyphenotypic small round cell tumor with an *EWS/ERG* fusion transcript. 

 Sorensen et al. [57] reported t(11;22) in 1 case of embryonal rhabdomyosarcoma and 4 cases of alveolar rhabdomyosarcoma. This raises questions about the genetic nature of so-called “primitive ectomesenchymomas”, lesions with a mixed myogenic-neural phenotype. These lesions are currently treated as rhabdomyosarcomas [58].

### 3.6. CD133 in Recurrent ESFT

In recent years, putative tumor-initiating cancer stem cells (tiCSC) have been isolated from human tumors [59]. TiCSC may be more resistant to standard chemo- and radiation-based therapies than bulk tumor cells [60]. CD133 has been described as putative marker of tiCSCs in ESFT [61]. It is thus proposed that CD133 may be a marker of chemoresistance in at least some cases of primary ESFT [61].

## 4. Cytogenetics and Molecular Features of ESFT

Specific balanced chromosomal translocations cytogenetically characterize ESFT [45] and create aberrant chimeric fusion oncogenes with different transactivation activities [62]. Currently, the gold standard for the diagnosis of ESFT is confirmation of histological diagnosis by cytogenetics/molecular studies. In general, tumors lacking genetic confirmation and signs of specific differentiation are termed “undifferentiated sarcomas” and are currently treated as nonrhabdomyosarcomatous soft tissue sarcomas by the Children's Oncology Group (COG) [63].

### 4.1. Translocations Involving EWS (See Table 3)

In 99% of cases, molecular fusions of ESFT involve the *EWS *gene (also known as *EWSR1*; located on chromosome 22) and a member of the ETS family of transcription factors, which includes *FLI1 *(on chromosome 11) and* ERG* (in chromosome 21). In 5%–10% of ESFT, *EWS* is fused to other ETS members such as *ETV1*,* ETV4*, or *E1AF* [64]. Although it is a promiscuous gene that creates chimeras in a variety of neoplasms, fusions of *EWS* to ETS family genes are unique to ESFT. The list of variants of ETS family that genes may substitute for *FLI1* continues to grow. Regardless of the ETS partner involved, variant translocations do not alter the tumor phenotype [65]. *EWS* fuses to non-ETS genes in other types of tumors, such as desmoplastic small round cell tumor, angiomatoid fibrous histiocytoma, clear cell sarcoma of soft tissue, and extraskeletal myxoid chondrosarcoma [66, 67]. Therefore, the partner gene, rather than *EWS per se*, appears to specify the tumor type.

Within the t(11;22), the chromosome breakpoint sites vary among 4 introns in *EWS* gene and six introns in the *FLI1* gene and yield a large number of possible *EWS-FLI1* fusion combinations [69]. *EWS-FLI1* is the most heterogeneous gene fusion in cancer [70]. Different combinations of exons from *EWS* and *FLI1 *create up to 18 possible types of in-frame *EWS-FLI1* chimeric transcripts [69]; these have been termed “type 1”, “type 2”, and so forth, fusions. Some reports indicate that fusion heterogeneity has functional and clinical significance [71]. Zoubek et al. [72] and De Alava et al. [73] reported that respective subgroups of 55 and 99 patients with localized ESFT and type 1 EWS-FLI1 fusions had longer relapse-free survival than those with nontype 1 fusions. Two more recent prospective studies [15, 72] however indicated that with more effective therapy there is no survival advantage of type 1 fusions in ESFT. 

 The alternate ESFT translocation t(21;22) causes a fusion of *EWS *to *ERG*, which encodes a transcription factor highly related to *FLI1*. *EWS/ERG *induces hematopoietic tumors in knock-in mice, suggesting that it can operate in targets other than mesenchymal cells [74]. 

With the t(2;22), t(7;22), and t(17;22) [70], *EWS *respectively fuses to *FEV*, *ETV1,* and *EIAF* (additional members of the ETS transcription factor family). These translocations occur only rarely and have not been the subject of detailed study. However, note that their presence would not be detected in ESFT if only *EWS/FLI1* and *EWS/ERG* RTPCR were performed.

In three reported cases of CD99-negative small round cell tumors resembling ESFT, *EWS* juxtaposes to *SP3, 2NF278*, and *POU5FI* genes, which encode transcription factors not been previously implicated in ESFT [75]. Although extremely rare, lesions with the morphology and phenotypic of polyphenotypic small round cell tumor, rhabdomyosarcoma, or desmoplastic small round cell tumor have been reported to contain either *EWS-FLI1* [56] or *EWS-ERG* fusion genes [76].

### 4.2. ESFT Translocations Not Involving EWS (See Table 3)

**Figure 4:**
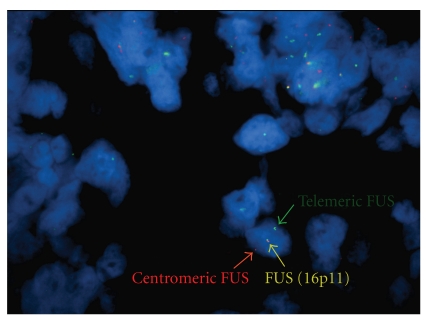
Ewing's sarcoma with *FUS* translocation. This CD99-positive lesion arose from the femur and had typical Ewing's sarcoma morphology. It was negative for an *EWS* rearrangement by both RT-PCR and FISH studies. In this interphase nucleus, note that one copy of the *FUS *locus has been divided, as evidenced by separate centromeric and telomeric gene signals, whereas the other copy has an intact, conjoined signal. Since breakapart FISH only tests a single gene locus, the partner gene in this translocation is unknown (fluorescence in situ hybridization of the *FUS* locus; courtesy of Dr. Ji-Yun Lee, University of Oklahoma).

Variant translocations involving the TET family to which *EWS* belongs have also recently been described. These “promiscuous” molecular partnerships may cause false-negative results during diagnostic evaluation if the appropriate probes are not used (Figure 4) [70]. *FUS* (also a member of the TET family of RNA-binding proteins) shows considerably homology to EWS and rarely substitutes for *EWS* in ESFT fusion formation (reviewed by Barr [70]). The resultant t(16:21)(p11;q24) produces an *FUS-ERG* fusion with no *EWS* rearrangement [77]. In the t(2;16)(q35;p11) balanced translocation, there is an in-frame fusion of *FUS* to *FEV *[78].

### 4.3. Translocation-Negative ESFT

Some ESFT-like cases with typical morphology and immunohistochemistry may be negative for the common translocations [70]. These lesions constitute genuinely challenging cases, as *EWS *fusion negativity casts doubts about the diagnosis. Many of these are being treated as “undifferentiated sarcomas” until new information can clarify their biologic nature. 

ESFT-like tumors showing similar morphology but lacking CD99 expression may have novel gene fusions. A completely novel gene fusion *CIL-DUX4* that does not involve genes related to *EWS* or ETS family members has been described in two cases [75]. The exact relationship of these lesions to ESFT is undetermined.

The growing complexity of ESFT-related genetic rearrangements indicates that individual cases may have ESFT morphology and phenotype but lack evidence of *EWS* fusions by standard methodology [70]. As noted above, both partner genes may be separately involved in variant translocations [77]. Thus, a negative result generated by genetics testing should not absolutely preclude a diagnosis of ESFT in the context of typical morphological and immunophenotypic features. This situation underscores the value of using classical karyotype analysis in sarcoma diagnosis, because of the ability of standard cytogenetics to interrogate the cancer genome for balanced chromosomal translocations and other genomic aberrations. On the other hand, one should remember that other round cell sarcomas, particularly synovial sarcoma, alveolar rhabdomyosarcoma, and T-cell lymphoma, may closely mimic ESFT and should be vigorously excluded in these situations.

## 5. Secondary Genetic Changes of ESFT

Recent studies have focussed on secondary changes of ESFT, such as mutations and epigenetic alterations, particularly as a means of predicting clinical outcome. This work detects molecular targets that could be used in high risk patients. However, most currently available studies suffer from limited sample size, overly complex amounts of data, and lack of multivariate analyses that consider clinical prognostic indicators such as site, age, and stage. Nevertheless, these recent reports offer exciting glimpses into future avenues for exploration and include studies at the DNA, RNA, and protein level.

### 5.1. Comparative Genomic Hybridization

Comparative genomic hybridization (CGH) has been used to detect numerical gene abnormalities in ESFT [79, 80]. Numerical abnormalities with gains in chromosomes 1q21-22, 8, and 12 have been reported [79, 80]. In a study of 62 ESFT by CGH, univariate analysis showed that patients with gains of chromosomes 1q, 2q, 12, and 20 or losses of 16q and 17p have significantly lower overall survival than those without such aberrations [81]. A separate CGH study by Savola et al. of 31 ESFT [82] reported that the most frequent copy changes were gains at 1q, 2, 8, and 12 and losses at 9p and 16q. In this study, patients whose tumors had three or fewer copy number changes had better survival than patients with tumors having a higher number of copy number aberrations. Expression array studies of 16 of Savola et al.'s cases identified 20 novel ESFT-associated oncogenes and tumor suppressor genes. This study adds new information regarding alterations in ESFT gene copy number and provides valuable data for future analyses.

### 5.2. Gene Expression Profiles

Other expression array studies have also yielded data of interest. Based on microarray study of a small population of 20 metastatic and primary ESFTs, Ohali et al. [83,] claimed that gene expression at diagnosis might be used to predict recurrence and metastases. In a similar study of 27 ESFT, Schaeffer [84] identified genes that were differentially regulated between metastatic and localized tumors and described characteristic gene expression signatures associated with metastases, including signalling pathways for activation of PDGF and WNT1, apoptosis, angiogenesis, alteration of p53, and resistance to chemotherapy.

The expression profile of *EWS/FLI* in cell lines has been modulated by use of inhibitory RNA segments (RNAi) [85–87]. This work identifies target genes of the fusion protein and suggests that the phenotype is mediated by the t(11;22)(q24;q12). With this model, there were 3-4 times as many genes that were down-regulated by EWS-FLI1 as were up-regulated. One target gene of EWS/FLI appears to be *NKX2.2*, whose expression is critical for the transformed phenotype of Ewing's sarcoma cells. *NKX2.2* plays a normal role in central nervous system and neuronal development, but its role in oncogenesis is unknown. 

Signalling [22] and metabolic pathways [88] are being actively investigated to identify potential candidates for therapeutic intervention and predictive prognostic factors.

### 5.3. Cell Cycle Genes

Studies involving translocations have generally overshadowed the relevance of secondary mutations in ESFT biology. However, genetic/epigenetic alteration of factors such as p53 or p16 may determine prognosis. p53 alterations appear to define a small clinical sunset of ESFT with a markedly poor outcome [89, 90]. Significant correlation exists between a good chemotherapeutic effect (as determined by tumor necrosis) and the absence of genetic alterations in p53 or p16/p14ARF [91]. p16 data appear contradictory. Loss of heterozygosity of the cell cycle regulatory gene *CDKN2A *(which codes for p16) has no prognostic significance in primary ESFT, suggesting no role for epigenetic modification of this gene [92]. High levels of p16/p14ARF mRNA predict poor event-free survival, but the results need further confirmation [92]. The inconsistency of data obtained by profiling p16 and p14ARF gene status, mRNA, and protein expression emphasizes the importance of investigating cell cycle regulatory genes in identical clinical samples, using standardized methods, and employing a prospective clinical outcome study. Data regarding p53 pathway impairment and poor ESFT prognosis suggests that this parameter should be investigated in future prospective studies to determine whether it should be added to routine staging analyses. Increased Ki-67 expression has also been reported as a valuable indicator of poor prognosis in localized ESFT [93].

### 5.4. Microsatellite Instability

Microsatellite instability (MI) has been of great interest in human cancer, particularly colon carcinoma. The prevalence of MI in ESFT and how this factor relates to prognosis are not clear. Aldinger et al. [94] retrospectively analyzed MI in ESFT and concluded that loss of mismatch repair protein expression is not prevalent in ESFT, but the nature of that instability differs from the form observed in colorectal carcinoma. Microsatellites have been found to be responsive elements to *EWS-FLI1* [22, 95, 96]. *EWS-FLI1* interacts with GGAA-microsatellites to regulate some target genes, including *NROB1*, an EWS-FLI1-regulated gene that is required for the oncogenic phenotype of ESFT [97].

## 6. Ontogenesis of ESFT

Since James Ewing in 1921 first described a diffuse hemangioendothelioma of bone [1], several studies have attempted to elucidate the histogenesis of ESFT (reviewed by Yunis [17]). Initial morphological and immunopathological data suggested that ESFTs arise from primary mesenchymal stem cells with potential for multilineage differentiation [40]. The expression of neural markers in ESFT suggests either a neuroectodermal origin or a potential mesenchymal cell origin with secondary expression of a partial neural phenotype.

Several recent articles focusing on expression profiles of ESFT cell lines have given new clues on molecular mechanisms related to the origin, development, and progression of ESFT [79–87]. A large number of EWS/FLI1 target genes have been identified, some of which are upregulated and others are downregulated.

Mesenchymal stem cells (MSCs) are currently considered strong candidates for the cell of origin of ESFT [20, 98, 99]. Among primary human cells, only MSCs display permissiveness for stable *EWS-FLI1* expression without undergoing growth arrest [20, 98]. In cell cultures, *EWS-FLI1 *behaves as an aberrant transcription factor that transforms MSCs by deregulating their gene expression [98]. In human pediatric MSCs (hpMSC), *EWS-FLI1* induces the expression of embryonic stem cell genes *OCT4, SOX2*, and *NANOG* [100] and numerous genes involved in neural and neuroectodermal differentiation. In special media, transfection of *EWS/FLI1 *and repression of the inhibitory effect of miRNA cause hpMSC to assume a neural crest stem cell phenotype and to generate a subpopulation of cells that display ESFT features [100]. The target gene *SOX2* appears to be a key player in determining ESFT cell differentiation and tumorigenicity [100].

The expression of neural markers in ESFT has also been intensely investigated. In 2005, Hu-liekovan [101] showed that EWS-FLI1 has a profound effect on cell differentiation as well as proliferation. They hypothesized that *EWS-FLI1 *acts as a lineage determinator rather than a pure oncogene, and they speculated that ESFT probably originates from primitive multipotent progenitor cells that are capable of differentiating into neural crest derivatives. *EWS-FLI1* subsequently may impose a neural crest parasympathetic direction to the cells, but it inhibits terminal differentiation. Eventual secondary genetic alterations may be the oncogenic events that lead to cancer. 

Baliko et al. in 2007 [102] showed that Notch signalling is active in ESFT tumors and cell lines, which express Notch receptors, ligands, and the Notch target gene *HES1*. Notch signalling inhibition causes ES cells to assume a more differentiated neuroid phenotype, also supporting the notion that ESFTs are derived from neuroectodermal precursors whose differentiation is inhibited by oncogenesis. Notch signalling inhibition induces neural differentiation but effects only minor changes in tumor growth, suggesting that it should not affect clinical outcome [29, 54, 103]. 

In 2010 [104] Gayscone reported that the neuronal marker BRN3A is expressed abundantly at the protein level in primary ESFT but not in neuroblastoma or rhabdomyosarcoma. Therefore, it appears that EWS/ETS proteins induce expression of neuronal markers and stimulate early stages of neuronal development but prevent terminal neuronal differentiation. EWS/ETS may regulate neuronal differentiation by controlling selective *BRN3A* transcriptional function.

## 7. Predictive Prognostic Factors in ESFT

A variety of predictive factors have been linked to survival of ESFT patients. These include clinical factors as well as the biological markers discussed above. The following section outlines our current understanding of prognostic factors and puts them into a clinical context.

### 7.1. Metastases

Presence of metastases is the most prominent adverse prognostic factor in ESFT; patients presenting with metastatic tumor have an estimated survival of only 20–25%. [19, 105–109]. Recurrence after therapy also portends a dismal outcome [110]. Recently, it has been reported that patients with primary disseminated multifocal ESFT may survive with intensive multimodal therapy [111].

Site of metastasis appears to be an important prognostic factor in MD. Patients with primary pulmonary metastases fare better than those with nonpulmonary tumor spread, especially following bilateral lung irradiation or myeloablative high-dose therapy (HDT) [107, 111–114], as compared to patients with primary bone and/or bone marrow involvement [108, 111, 113, 115–117]. 

In the Euro-Ewing 99 trial for patients with disseminated multifocal ESFT (excluding those with isolated pulmonary metastases) [111], increased risk was associated with the number of skeletal metastatic lesions. Of patients with more than 5 metastatic lesions at diagnosis, only 16% survived.

### 7.2. Localized Disease

Because of their higher survival rate, patients with localized disease at diagnosis offer a better opportunity to study prognostic factors in ESFT. In these patients, cooperative group and single institution studies have associated adverse outcome with older age at presentation (≥14 years [114, 118] or ≥18 years [106]), large tumor volume [119], poor response to induction therapy [120], axial location [114, 120], elevated LDH [121], secondary genetic abnormalities [122], deletion of p16 [123], and mutation of P53 [89, 90]. Prediction results vary widely among studies; they used different criteria (e.g., a large tumor may be defined as one >100 mL, >200 mL, or >8 cm) and treatments.

### 7.3. Age

The impact of age on the prognosis of ESFT remains controversial [124]. In different studies, the cut off for age analysis is variable: 14 [114, 117], 15 [108], or 18 years [106]. Ferrari [125] stratified ages as up to 14 y, from 15 to 18 y, and >18 y. In certain series, older age has been associated with inferior clinical outcome [106–108, 126], yet others have been unable to demonstrate a significant difference based on age alone [114, 124, 127, 128]. The impact of age can be confounded by the greater proportion of large pelvic primary tumours and more advanced disease in adult patients, and specific biological differences may also play a role [108, 124]. Older age remains an adverse prognostic factor despite the addition of ifosfamide and etoposide to the therapeutic regimen [106]. In a study to assess the impact of different therapies in children and adults, Gupta et al. [124] reported that adults with localized ESFT had an inferior outcome compared to pediatric patients, possibly due to dosage of alkylating agents and timing of local therapy.

### 7.4. Gender

Gender of patients is not significantly related to ESFT survival [106, 129].

### 7.5. Size (Tumor Volume)

Classically, large tumors have been associated with a worse prognosis. The size of the tumors has been assessed by longitudinal and volumetric measurements. Tumors greater than 8 cm are associated with a worse prognosis by univariate analysis [118, 130]. However, tumor size disappeared as a prognostic factor in the more intensive EW92 protocol [131]. In the CESS81 study, tumors greater than 100 cm^3^ were originally associated with worse outcome by univariate analysis [115]. However, with subsequent treatment improvements, the tumor size associated with a worse prognosis increased to greater than 200 cm^3^ [119]. In a multivariate analysis, Lee [129] reported that metastatic disease and large tumor size remained independent predictor factors, while pelvic involvement did not.

EICESS-92. the largest attempt to stratify ESFT therapy on the basis of tumor size [132] found no difference in survival between standard risk (localized small tumors) and high risk (large localized tumors or patients with metastatic disease) patients receiving standard therapy. Patients in the high risk arm with large localized tumors showed a trend toward improvement with the addition of etoposide, but this trend did not reach statistical significance [132, 133]. Because patients with metastases were in the same risk group as those with large tumors, the results cannot be interpreted based on size alone [133].

Primary tumor volume is a strong independent prognostic factor, even in patients with primary disseminated multifocal ESFT, and is more important than site of primary tumor. A trend toward worse outcome for central tumors has been seen, which may reflect problems in local control [111]. Larger tumor size will always represent a therapeutic challenge because of the problems with local control. However, without more standardized criteria for assessment of tumor size (longitudinal versus volumetric measurements), the value of volume as a prognostic factor will be imperfect [118].

### 7.6. Site

Axial tumors showed more aggressive behaviour in the early IESS (IESS-I) [134] and UKCCSG/MRC (ET-1) [107] studies. However, the same cooperative groups reported minimal or insignificant differences in outcome based on site (IESS-II and ET-2) [118, 135, 136]. The first POG-CCG Ewing trial (POG-8850/CCG-7881) reported that addition of ifosfamide-etoposide to the standard VACD regimen abrogated the negative prognostic implications of large tumor size (>8 cm) and pelvic location [118, 137]. Multivariate analysis has been controversial. Some studies have found pelvic location and time to local therapy to be associated with outcome [124], while other studies [129] reported that pelvic involvement was not independently associated with risk of death. 

The increased prevalence of pelvic ESFT in adult patients is often cited as one of the main contributors to poor outcome [124]. It is likely that pelvic tumors are diagnosed later than tumors of the extremities because of subclinical extension into the pelvic cavity. This factor is consistent with the greater tumor volume of pelvic tumors and their significantly higher proportion of metastases at diagnosis [109, 129].

### 7.7. Neural Differentiation

The effect of neural differentiation on ESFT behaviour has been a source of conflicting results. For this purpose, ES must be consistently separated from pPNET, but tumoral heterogeneity makes this a difficult task. In spite of initial enthusiasm for neural differentiation as a prognostic factor (reviewed by Dehner [16]), subsequent retrospective studies have found it has no significant impact on clinical behaviour [28, 29, 103, 138]. 

Of interest, however, is the observation that outcome of osseous and extraosseous ESFTs did not differ in a recent Children's Oncology Group study [139]. An unpublished analysis of osseous ESFT patients by the Children's Cancer Study Group and Pediatric Oncology Group found that in nonmetastatic patients, neural differentiation even offered a survival advantage [140]. Similarly, Parham et al. [54] in 1999 found that neuroectodermal differentiation did not predict tumor behaviour in a retrospective study of 60 cases treated by modern therapy in two large institutions. They suggested that with improved multiagent chemotherapy, surgery, and radiation, differences in outcome between pPNET and ES no longer exist. This was also been reported in other studies [103]. Because of these conflicting results, current studies no longer separate ES from pPNET for prognostication or treatment. 

However, Bacci et al. in 2000 [141] indicated that patients with PNET of bone fare significantly worse than patients with classical ES. Of note, cytogenetic results were not included in this study, and the same authors made no claims regarding neural differentiation in two subsequent outcome analyses of nonmetastatic ESFT [141, 142].

Atypical ESFT has been recently claimed in retrospective study to be the only histological variety associated with a less favourable clinical outcome [24], although this was not the case in the initial reports [10, 11].

### 7.8. Chemotherapy Response

During the past 30 years, improvements in systemic and local therapies have increased survival from less than 5% overall to 65%–70% for localized ESFT and to 25%–30% for metastatic tumors [114, 118, 143]. Alkylating agents (ifosfamide and cyclophosphamide) and anthracyclines (doxorubicin) are the two groups of agents most effective against ESFT. High-risk patients seem to have benefited from intensified treatment that incorporated ifosfamide [106, 117, 119]. 

 Current chemotherapy regimens use the same drugs (vincristine, doxorubicin, cyclophosphamide, ifosfamide, and etoposide), although the exact regimen differs between Europe and North America [19, 105, 144]. Chemotherapy dose intensification and the use of megatherapy with autologous hematopoietic stem cell transplant seem valid alternatives for high risk patients. 

Many factors can affect the feasibility, modalities, and timing of proposed treatments. Factors affecting the pharmacokinetics of cancer chemotherapy have important consequences in terms of therapeutic efficacy and safety. Cytochrome P450 [145] plays an important role in the biotransformation of anticancer agents [146] and metabolizes cyclophosphamide and ifosfamide. Factors such as age, genetic polymorphism, and intake of drugs and food can modify the activity of cytochrome P450, [147]. An influence of age on pharmacokinetics of ifosfamide has been reported [148]. 

Scotlandi (2009) [149] analysed the molecular factors that reflect tumor resistance to chemotherapy in ESFT and found that glutathione metabolism is a major pathway regulating ESFT chemoresistance. Glutathione S-transferases (GSTs) are a family of detoxification enzymes that catalyze the conjugation of glutathione to a wide variety of endogenous and exogenous compounds. GSTs have been implicated in the development of resistance toward chemotherapeutic agents [150]. Low expression of MGST1 (microsomal glutathione S-transferase 1) is significantly associated with better prognosis.

### 7.9. Chemotherapy-Induced Necrosis

In patients treated with primary chemotherapy followed by surgical excision, assessment of chemotherapy-induced necrosis allows reliable evaluation of tumor chemosensitivity. Good histological response is strongly related to good clinical outcome [119, 125, 126, 128]. The degree of necrosis seems to be independent of the drugs used and the length of preoperative chemotherapy [125]. Histological response is determined by the method of Salzer-Kuntschick et al. [151], in which 10% or fewer viable tumor cells in the surgical specimen are classified as “good response” and greater that 10% viability is regarded as “poor.” Ferrari et al. (2007) [125] investigated the influence of traditional factors (age, sex, site and size of the tumor, fever, and lactate dehydrogenase levels) on histological response of nonmetastatic ESFT; age and sex appear to influence the degree of necrosis.

## 8. Conclusions

Progress in the treatment of ESFT in the last three decades has derived largely from American and European cooperative trials using chemotherapy intensification and improved local control [19, 105, 118, 152]. Chemotherapy intensification and optimal local control have overcome differences in classical clinical, and biological prognostic factors that formerly portended a poor outcome [105]. With most modern treatment regimens, the disease-free survival for patients with localized disease may approach 70%, while overall survival may exceed 80% [19, 105, 117, 118]. However, a 30% relapse rate is still unacceptably high, considering that most relapsed patients do not survive [153]. 

The improvement of ESFT therapy is linked to the discovery of new strategies to select patients with poor and good prognosis. In the COG studies in North America, there are 3 risk groups: patients with localized tumours, patients with lung metastases only, and patients with other multiple metastases. Euro E.W.I.N.G. 99 study uses metastatic sites (none, lung, or other) and initial tumor size (< or > than 200 mL) for initial stratification and subsequently considers resectability and histological response to initial chemotherapy to assign patients to treatment randomization [144]. Therefore, excepting metastases at diagnosis, tumor burden, and response to chemotherapy, little is known about factors that determine disease progression or relapse [19]. 

Drug-resistant disease remains a major cause of mortality among patients diagnosed with ESFT. An improved understanding of the mechanisms of drug resistance and discovery of biomarkers associated with chemoresistance are needed for improved treatment of ESFT patients. Molecular signatures [149] offer a novel means of classification of ESFT patients into high- and low-risk groups, The glutathione metabolism pathway has emerged as one of the most significant pathways associated with prognosis. A small molecule inhibitor of GST enzymes 6-(7-nitro-2, 1, 3-benzoxadiazol-4-ylthio) hexanol NBDHEX has been proposed as a new potential therapeutic agent [149, 154].

The elucidation of the cell of origin is a crucial issue for discovering the mechanisms involved in the genesis of ESFT and for identification of reliable molecular markers and possible therapeutic agents. Mesenchymal stem cells or progenitor cells are currently considered strong candidates as the cell of origin of ESFT [20, 98, 99]. 

Preliminary data suggest that assessment of CD133 or Ki67 expression in diagnostic biopsies may identify tumors with poor outcome [61, 93], but confirmation is needed. The claim that atypical ES is associated with less favourable outcome needs to be tested in future trials, using clear-cut, reproducible criteria. The pathological criteria to differentiate ES versus pPNET also need to be clearly defined, so that we can establish strict criteria for diagnosis that permit reliable assessment of these features. Future research in the histopathological characterization of ESFT is thus warranted. 

Efforts are ongoing to define the mechanisms of tumorigenesis molecular interactions of EWS/FLI1. Target genes that may be accessible for therapeutic agents are receptor tyrosine kineses, histone deacetylases, heat shock proteins, and mTOR derivatives [144, 153, 155].

Secondary mutations that could be of relevance in the biology of ES have often been usually overshadowed by studies of translocations. Secondary events could have relevance not only for prognosis but also for understanding the steps involved in cell transformation. Losses of p161*INK4A*, mutation of *TP53*, and deletion of *CKDN2A* appear to correlate with a poor prognosis [90, 91, 123]. Several groups [22, 115, 156] have identified gains and losses of various chromosomes that may be associated with a poor prognosis [82]. However, none of these parameters has been tested prospectively in a meaningful cohort of patients. 

In summary, a better understanding of the molecular pathogenesis and biology of ESFT is leading to a new definition of potential targets for antitumour therapy. As the regulatory pathways responsible for transformation, growth, and metastases of ESFT become more defined, potentials for new therapeutic targets will expand. This situation has created both a challenge and an opportunity to develop predictive biomarkers capable of selecting patients most likely to benefit from targeted therapy.

## Figures and Tables

**Table 1:** Morphological patterns described in two recent reviews of ESFT [23, 24].

Number of cases	Classical	pPNET	Atypical	Spindle	Sclerosing	Adamantinoid	Reference
66	46	9	3	3	2	3	[23]
415	280	53	82	nr	nr	nr	[24]

Nr: not reported.

**Table 2:** Immunohistochemical expression of ESFT markers.

Reference	CD99	FLI1	CK (AE1/3)	HMWCK	desmin	CD117	CAV1	CD57 (HNK1)
Folpe et al. [23]	100%	94%	32%	5%	2%	24%	ND	ND
Llombart-Bosch [24]	99%	89%	ND	ND	ND	ND	96%	53%

ND: not done; CK: cytokeratin; HMWCK: high molecular weight cytokeratin; CAV: caveolin 1; HNK1: human natural killer antigen 1.

**Table 3:** Translocations reported in ESFT [22, 67, 68].

Translocation	Fusion gene	Per cent positive
t(11;22)(q24;q12)	*EWS-FLI1*	>85
t(21;22)(q22;q12)	*EWS-ERG*	5–10
t(19;der)inv(21;22)	*EWS-ERG*	<1
t(7:22)(p22;q12)	*EWS-ETV1*	<1
t(17;22)(q12;q12)	*EWS-ETV4*	<1
t(2;22)(q33;q12)	*EWS-FEV*	<1
t(6;22)(p21;q12)	*EWS-POU5F1*	<1
t(1;22)(q36.1;q12)	*EWS-PATZI*	<1
t(2;22)(q31;q12)	*EWS-SP3*	<1
t(20;22)(q13;q12)	*EWS-NFATc2*	<1
t(16;21)(p11;q22)	*FUS-ERG*	<1
t(2;16)(q35;p11)	*FUS-FEV*	<1
t(4;19)(q35;q13)	*CIC-DUX4*	<1
